# Associations between health culture, health behaviors, and health-related outcomes: A cross-sectional study

**DOI:** 10.1371/journal.pone.0178644

**Published:** 2017-07-26

**Authors:** Yingnan Jia, Junling Gao, Junming Dai, Pinpin Zheng, Hua Fu

**Affiliations:** 1 School of Public Health, Key Lab of Public Health Safety of the Ministry of Education, Fudan University, Shanghai, China; 2 Health Communication Institute, Fudan University, Shanghai, China; Mexican Social Security Institute, MEXICO

## Abstract

**Background:**

To examine the associations between demographic characteristics, health behaviors, workplace health culture, and health-related outcomes in Chinese workplaces.

**Methods:**

A total of 1508 employees from 10 administrative offices and 6 enterprises were recruited for a cross-sectional survey. Self-administered questionnaires mainly addressed demographic characteristics, health behaviors, workplace health culture, and health-related outcomes including self-rated health, mental health, and happiness.

**Results:**

The proportion of participants who reported good health-related outcomes was significantly higher in those working in administrative offices than those working in enterprises. The result of the potential factors related to self-rated health (SRH), mental health, and happiness by logistic regression analyses showed that age and income were associated with SRH; type of workplace, age, smoking, and health culture at the workplace level were associated with mental health; and beneficial health effects of direct leadership was positively associated with happiness. Moreover, there were some similar results among 3 multivariate regression models. Firstly, good SRH (Odds Ratio (OR) = 1.744), mental health (OR = 1.891), and happiness (OR = 1.736) were more common among highly physically active participants compared with those physical inactive. Furthermore, passive smoking was negatively correlated with SRH (OR = 0.686), mental health (OR = 0.678), and happiness (OR = 0.616), while health culture at the individual level was positively correlated with SRH (OR = 1.478), mental health (OR = 1.654), and happiness (OR = 2.916).

**Conclusions:**

The present study indicated that workplace health culture, health behaviors, and demographic characteristics were associated with health-related outcomes. Furthermore, individual health culture, physical activity, and passive smoking might play a critical role in workplace health promotion.

## Introduction

Health culture can be seen as a social norm that values health as the nation’s priority or as an appeal to improve the social determinants of health [1]. In the past, people do not have much chance to choose their lifestyle under low living standards. Thus, the role of health culture was not obvious. However, as living standards improve, the effect of health culture has become increasingly important because people have begun to conditionally determine the use of resources and decided their lifestyle [2]. Culture might affect people's thinking and behavior, thereby affecting health. Workplaces are more likely to foster their own culture with certain characteristics than others. Workplace health culture consists of three levels including individual, direct leaders, workplace [3]. Therefore, workplace health culture mainly focus on employees’ health beliefs and health norms, leaders’ health behaviors and effect, and health related policy and value. In 2010, WHO proposed a Healthy Workplace Model based on their identified needs: health and safety concerns in the physical work environment; health, safety, and well-being concerns in the psychosocial work environment, including the organization of work and workplace culture; personal health resources in the workplace (support and encouragement of a healthy lifestyle by the employer); and ways of participating in the community to improve the health of workers, their families, and members of the community. The Healthy Workplace Model helped clarify the factors influencing the health of the working population [4]. Workplace health culture was especially highlighted based on the Healthy Workplace Model. Several researchers have begun to focus on the impact of workplace culture: Through a cross-sectional survey of 349 employees at three separate furniture-manufacturing facilities, Hall et al. [5] found that the better the workplace culture, the higher participation rate in health promotion programs. Golaszewski et al. [6] presented a conceptual model for addressing the creation of supportive environments for worksite health promotion settings, and emphasized the importance of improving the cultural environment of workplaces for health promotion. Results of a qualitative study conducted in-depth interviews with representatives from 31 organizations representing small, medium and large businesses in Western Australia showed that improving health culture had an important impact on health promotion [7]. However, most existing studies lack support of convincing quantitative data [8]. Although Kwon et al.[8] had developed a scale to measure workplace culture of health, existing studies failed to examine the associations between health culture and health outcome by convincing quantitative data. Therefore, how about the impact to health outcome from the health culture in China is still unclear. Moreover, considering the diversity and complexity of health culture, its impact on physical health and mental health may be different. Therefore, we have measured three health outcomes including self-rated health, mental health, and happiness in the study. In order to more scientifically evaluate the impact of health culture on different health outcomes, it is necessary to understand the influencing factors of health outcomes based on previous studies.

In terms of self-rated health (SRH), a cross-sectional study of Chinese employees showed that SRH was associated with demographic characteristics, psychosocial work environment, and lifestyles such as physical activity and passive smoking [9]; another cross-sectional study in Southern Europe found that younger participants, males, higher educated participants, and participants with lower body mass index had more chances than older, less educated, and higher body mass index participants, respectively, to report better SRH [10]; the results of a survey in Mozambique showed that SRH was associated with gender, age, marital status, and physical activity [11]; a study of the Arab population in Israel found that higher education level and current employment in old age were associated with better SRH, and greater physical activity was found to be related to good/very good SRH, while obesity was associated with less than good SRH [12]. Regarding mental health and happiness, Stickley et al. [13] found that ex-smokers and those who have never smoked were significantly happier than current smokers. Furthermore, Kobau et al.[14] emphasized that well-being differed by demographic characteristics such as marital status, health behaviors, chronic conditions, and disability status. Additionally, a community-based intervention study highlighted that positive lifestyle changes such as increasing physical activity levels and increasing fruit and vegetable consumption were associated with positive changes in mental health [15]. So SRH and well-being were considered as indicators of health outcome by many studies.

Considering the differences in the concepts of workplace health culture between Western countries and China, a Chinese Workplace Health Culture Scale (WHCS) was developed, and showed good reliability and validity [3]. By applying the Chinese WHCS, this study is the first to quantitatively evaluate workplace health culture, and examine the associations between demographic characteristics, health behaviors, workplace health culture, and health-related outcomes in 10 administrative offices and 6 enterprises. To provide a more comprehensive assessment of the health status of participants, 3 health variables including SRH, mental health, and happiness were measured in this study as the primary outcomes.

## Method

### Informed consent form

Written informed consent statement forms were obtained from participants. The right to withdraw and autonomy of responses were also explained. This study received ethical approval from the ethics committee of School of Public Health of Fudan University, China.

### Data collection

Each interviewer was responsible for collecting the self-administered questionnaires from each worksite during the working day from July to November 2014 in Shanghai. Questionnaires were returned from all selected 16 workplaces and most selected employees. Of the 1600 employees who were administered the survey, 1508 (94.3%) returned a completed survey. Participants were recruited from 2 representative types of workplaces: 10 administrative offices at primary level of government and 6 enterprises. The questionnaire included 4 parts: (1) demographic characteristics, (2) health-related outcomes, (3) health behaviors, (4) WHCS. Please refer to S1 and S2 Files for details of the survey.

### Demographic variables

Self-reported demographic variables included type of workplace, gender, age, marital status, education, and family per-capita monthly income. Age, education, and income were each separated into 4 categories. The 4 age categories were <30, 30–39, 40–49, and ≧50 years. The 4 education categories were junior high school, high school/technical secondary school, junior college, and bachelor’s or higher university degree. The 4 categories for income were <¥2000, ¥2000–3999, ¥4000–5999, and ¥6000.

### Measurements

#### SRH

SRH was one of the main health-related outcomes in our study. SRH is generally assessed by a single survey question inviting participants to provide a subjective assessment of their health using some form of a five-point scale [16]. Participants were asked to rate their own general health ranging from perfect to poor by answering “would you say that in general your health is perfect, very good, good, fair, or poor?”. As a dependent variable in logistic regression, the original variable was dichotomized according to the distribution of SRH, with 1 representing perfect, very good, and good health, and 0 representing fair and poor health.

#### Mental health

As another main health-related outcome, mental health was measured by the Chinese version of WHO-Five Well-being Index. The WHO-5 has demonstrated excellent psychometric properties in a large representative sample [17, 18]. We used 5 questions to investigate the status of respondents over the past 2 weeks. For instance, how often have you felt cheerful and in good spirits [17]? We used 5 questions to investigate the status of respondents over the past 2 weeks. Respondents answered each question on a 6-point scale ranging from never (0) to all the time (5). According to the total scores <13 points or ≥ 13 points, the respondents were divided into "poor mental health" or "good mental health", respectively.

#### Happiness

Another main health-related outcome, happiness was measured by the Chinese version of the Flourishing Scale (FS) which showed good validity and internal consistency with a Cronbach alpha coefficient of 0.86 [19]. The FS consisted of 8 items, each measuring a core aspect of optimal social-psychological functioning on a 7-point Likert scale ranging from 1 (strongly disagree) to 7 (strongly agree) [19]. As a dependent variable in logistic regression, the original variable was dichotomized based on the distribution of happiness. According to the average scores <5 points or ≥5 points, respondents were divided into "poor happiness" and "good happiness," respectively.

#### Health behaviors

Smokers were classified as respondents who had smoked more than 100 cigarettes [20]. Passive smokers were respondents who had been exposed to others’ smoke for more than 15 minutes in the last week [21]. Alcohol intake was dichotomized with 1 representing yes, and 0 representing no. The self-reported data for physical activity was collected from the Chinese version short International Physical Activity Questionnaire (IPAQ), which was acceptably reliable (intraclass correlation coefficient of 0.79) [22]. In brief, the IPAQ short form asked about 3 specific types of physical activity: walking, moderate-intensity activities, and vigorous-intensity activities. According to IPAQ guidelines, physical activity level was divided into 3 categories: high, moderate, and low. More details about IPAQ can be found in the literature [9].

#### Workplace health culture

Workplace health culture was measured as an individual-level variable. Based on the Chinese situation, we developed a WHCS. A convenient sample of 976 employees from 12 workplaces was investigated. Results showed good construct validity and content validity. The Cronbach’a coefficient of all the dimensions ranged 0.724–0.908, which indicated good reliability [3]. There were 20 items in the scale mainly measuring health culture, which were divided into 5 dimensions: individual health culture, adverse health behaviors of direct leaders, adverse health effects of direct leaders, beneficial health effects of direct leaders, and overall health culture [3]. The 20 Likert-scale items of the WHCS are presented in Table 1. Each item ranged from 1 to 5, in which a higher score indicated better workplace health culture. As a continuous variable, the average score of each dimension was calculated respectively, which was then included in subsequent analysis.

**Table 1:** Items of Chinese Workplace Health Culture Scale.

Variable	Item
Individual health culture	1. It is important for me to lead a healthy lifestyle
	2. Participation in health activities can enlarge my circle of friends
	3. Employee will be commended and paid attention to due to healthy behaviors
	4. My family or roommates support me to lead a healthy lifestyle
Adverse health behaviors of direct leaders	5. My direct leaders like smoking
	6. My direct leaders like drinking
Adverse health effects of direct leaders	7. My direct leaders encourage me to smoke
	8. My direct leaders encourage me to drink
	9. My direct leaders hope that I can work overtime
beneficial health effects of direct leaders	10. My direct leaders like to exercise
	11. My direct leaders encourage me to exercise
	12. My direct leaders encourage me to lead a healthy lifestyle
	13. My direct leaders support each other to lead a healthy lifestyle
Overall health culture	14. Resources are provided to support health promotion.
	15. Employee are taught to lead a healthy lifestyle
	16. New employee notice organizational support on health behaviors
	17. Unhealthy behaviors are not encouraged
	18. People have team spirit
	19. A consensus has been reached.
	20. People have positive perception

### Data analyses

Descriptive analyses, analyses of variance, and chi-squared tests were conducted to compare the differences of health-related outcomes among different groups. Multiple logistic regression analyses were conducted to examine whether the factors including demographic variables, health behaviors, and workplace health culture influenced SRH, mental health, and happiness. For all analyses, statistical significance was set at 0.05. Statistical analysis was performed using the Statistical Package for Social Sciences 20.0.

## Results

The demographics of the study sample by different health-related outcomes are reported in Table 2. The final sample consisted of 1,508 participants (64.7% from administrative offices and 35.3% from enterprises). Nearly 60% of participants were 30 to 49 years old. Nearly 85% were married, and over half had a bachelor’s or higher degree. Additionally, over 60% of participants reported that their family per-capita income ranged from 2000–5999 RMB/month. The proportion of participants who reported good SRH was significantly higher in those working in administrative offices than that of those working in enterprises. Similar results were found in both mental health and happiness. Furthermore, we found significant differences in the distribution of SRH and mental health among different groups of age: the proportion of young participants (<30 years) reporting good SRH was highest, while the proportion of old participants (≧50 years) reporting good mental health was highest; the proportion of married participants who reported good happiness was higher than that of unmarried participants; the proportion of participants with a bachelor’s or higher degree who reported good SRH and happiness was both highest; and the proportion of individuals who reported good SRH increased significantly with income.

**Table 2:** Demographics of the participants by different health-related outcomes.

Variables		Overall *n* (%)	Good SRH *n* (%)	Fair or poor SRH *n* (%)	χ^2^(P)	Good Mental health *n* (%)	Poor Mental health *n* (%)	χ^2^(P)	Good happiness *n* (%)	Poor happiness *n* (%)	χ^2^(P)
Type of workplace	Government	975(64.7)	743(76.7)	226(23.3)	**14.472 (<0.001)**	824(84.9)	146(15.1)	**23.373 (<0.001)**	804(83.0)	165(17.0)	**36.999 (<0.001)**
	Enterprises	533(35.3)	359(67.6)	172(32.4)		391(74.8)	132(25.2)		359(69.3)	159(30.7)	
Gender	Male	736 (48.9)	546(74.7)	185(25.3)	1.231 (0.267)	601(82.7)	126(17.3)	1.517 (0.218)	552(76.3)	171(23.7)	2.900 (0.089)
	Female	768 (51.1)	552(72.2)	213(27.8)		611(80.2)	151(19.8)		608(80.0)	152(20.0)	
Age, years	<30 yr	287 (19.5)	237(82.6)	50(17.4)	**17.371 (0.001)**	230(81.0)	54(19.0)	**13.400 (0.004)**	219(76.8)	66(23.2)	3.612 (0.306)
	30–39 yr	506 (34.3)	363(72.2)	140(27.8)		391(77.6)	113(22.4)		381(75.9)	121(24.1)	
	40–49 yr	369 (25.0)	251(68.6)	115(31.4)		296(80.9)	70(19.1)		286(78.4)	79(21.6)	
	≥50 yr	313 (21.2)	224(72.0)	87(28.0)		269(87.9)	37(12.1)		246(81.5)		
Marital status	Married	1179(84.6)	860(73.4)	311(26.6)	1.261 (0.261)	955(81.8)	212(18.2)	0.007 (0.933)	925(79.6)	237(20.4)	**4.827 (0.028)**
	Unmarried/Divorced/Widowed	214(15.4)	165(77.1)	49(22.9)		174(82.1)	38(17.9)		156(72.9)	58(27.1)	
Education	Junior high school	85 (5.7)	58(69.9)	25(30.1)	**17.536 (0.001)**	67(82.7)	14(17.3)	0.737 (0.864)	64(80.0)	16(20.0)	**9.199 (0.027)**
	High School/technical secondary school	198 (13.3)	141(72.7)	53(27.3)		164(83.2)	33(16.8)		147(75.0)	49(25.0)	
	Junior college	348 (23.4)	226(65.3)	120(34.7)		278(80.6)	67(19.4)		251(73.2)	92(26.8)	
	Bachelor’s or higher degree	858 (57.6)	660(76.9)	198(23.1)		691(81.1)	161(18.9)		685(80.6)	165(19.4)	
Family per-capita monthly income (RMB)	~2000	161(11.0)	103(64.4)	57(35.6)	**15.807 (0.001)**	124(77.5)	36(22.5)	5.745 (0.125)	119(74.8)	40(25.2)	3.687 (0.297)
	2000~	475(32.6)	330(69.8)	143(30.2)		372(79.0)	99(21.0)		361(77.1)	107(22.9)	
	4000~	418(28.6)	322(77.0)	96(23.0)		349(84.3)	65(15.7)		324(78.3)	90(21.7)	
	6000~	405(27.8)	313(77.3)	92(22.7)		329(82.0)	72(18.0)		327(81.3)	75(18.7)	

Table 3 shows that most health behaviors, and health culture, were significantly associated with health-related outcomes. The proportion of individuals who reported good SRH, mental health, and happiness increased significantly with physical activity level, respectively. Similar results were found between passive smoking and health-related outcomes. The proportion of smokers who reported good SRH was significantly lower than that of non-smokers.

**Table 3:** Comparison of health-related outcomes by different health behaviors and health culture.

Variables		Overall *n* (%)	Good SRH *n* (%)	Fair or poor SRH *n* (%)	χ^2^(P)/F(P)	Good Mental health *n* (%)	Poor Mental health *n* (%)	χ^2^(P)/F(P)	Good happiness *n* (%)	Poor happiness *n* (%)	χ^2^(P)/F(P)
Physical activity	High	275(18.5)	221(80.7)	53(19.3)	**17.583 (<0.001)**	239(87.9)	33(12.1)	**18.322 (<0.001)**	230(84.6)	42(15.4)	**14.713 (0.001)**
	Moderate	700(47.1)	525(75.1)	174(24.9)		581(83.1)	118(16.9)		556(79.9)	140(20.1)	
	Low	510(34.3)	340(67.5)	164(32.5)		382(76.1)	120(23.9)		365(73.3)	133(26.7)	
Smoking	Yes	359(23.9)	248(69.1)	111(30.9)	**4.803 (0.028)**	281(78.9)	75(21.1)	1.790 (0.181)	264(75.0)	88(25.0)	2.686 (0.101)
	No	1144(76.1)	852(74.9)	285(25.1)		931(82.1)	203(17.9)		895(79.1)	236(20.9)	
Passive smoking	Yes	673(45.4)	458(68.2)	214(31.8)	**17.798 (<0.001)**	511(76.6)	156(23.4)	**19.027 (<0.001)**	482(72.6)	182(27.4)	**21.927 (<0.001)**
	No	811(54.6)	627(77.9)	178(22.1)		689(85.5)	117(14.5)		662(82.8)	138(17.3)	
Alcohol intake	Yes	440(29.5)	325(74.5)	111(25.5)	0.332 (0.564)	353(81.5)	80(18.5)	0.014 (0.906)	337(77.5)	98(22.5)	0.196 (0.658)
	No	1052(70.5)	766(73.1)	282(26.9)		850(81.3)	196(18.7)		815(78.5)	223(21.5)	
Workplace health culture	Health culture at individual level	4.38±0.67	4.45±0.65	4.21±0.69	**34.031 (<0.001)**	4.44±0.65	4.11±0.70	**55.107 (<0.001)**	4.53±0.55	3.85±0.78	**307.195 (<0.001)**
	Unhealthy behaviors of direct leadership	3.74±1.35	3.74±1.38	3.74±1.26	0.000 (0.983)	3.76±1.37	3.67±1.25	0.833 (0.361)	3.79±1.38	3.59±1.21	**5.483 (0.019)**
	Adverse health effects of direct leadership	3.97±1.18	3.96±1.22	3.98±1.07	0.055 (0.815)	3.96±1.21	4.01±1.06	0.455 (0.500)	4.01±1.21	3.82±1.05	**6.525 (0.011)**
	Beneficial health effects of direct leadership	4.12±0.84	4.18±0.83	3.96±0.85	**20.024 (<0.001)**	4.19±0.83	3.82±0.82	**43.894 (<0.001)**	4.28±0.78	3.57±0.80	**199.971 (<0.001)**
	Health culture at workplace level	4.15±0.82	4.22±0.81	3.99±0.82	**23.299 (<0.001)**	4.23±0.79	3.83±0.86	**55.126 (<0.001)**	4.31±0.75	3.61±0.82	**207.389 (<0.001)**

For workplace health culture at the individual level, the scores of participants who reported good SRH, mental health, and happiness was significantly higher than those who did not. Similar results were found in both beneficial health effects of direct leaders and health culture at the workplace level. Lastly, participants who reported good happiness had significantly higher scores for unhealthy behaviors of direct leaders and adverse health effects of direct leaders than other participants.

We then conducted logistic regression analyses to identify the potential factors that influenced SRH, mental health, and happiness (Table 4). The dependent variables for regression logistic model A, B and C were SRH, mental health, and happiness, respectively. In model A, participants <30 years old were more likely to have good SRH (Odds Ratio (OR) = 2.022) than participants >50. Participants whose family per-capita monthly income <2000 or ranged from 2000 to 3999 had 0.516 or 0.635 times lower odds of reporting good SRH compared with those with an income >6000 RMB. In model B, participants from administrative offices were more likely to have good mental health than those from enterprises. Compared with participants >50 years old, employees from the other 3 age groups had 0.521, 0.451, and 0.547 times lower odds of reporting good mental health. Furthermore, smokers had 0.583 times lower odds of reporting good mental health, and health culture at the workplace level was positively correlated with mental health (OR = 1.368). In model C, the beneficial health effects of direct leaders were positively associated with happiness. Moreover, there were some similar results among the 3 models. Firstly, good SRH, mental health, and happiness were more common (OR = 1.744, 1.891, and 1.736, respectively) among highly physically active participants compared with those physical inactive. Additionally, passive smoking was negatively correlated with SRH (OR = 0.686), mental health (OR = 0.678), and happiness (OR = 0.616), while health culture at the individual level was positively correlated with SRH (OR = 1.478), mental health (OR = 1.654), and happiness (OR = 2.916).

**Table 4:** Odds ratios (OR) and 95% confidence intervals (CI) of predictors of good health-related outcomes.

	Model A: *n* = 1249 Good self-rated health	Model B: *n* = 1246 Good mental health	Model C: *n* = 1245 Good happiness
Variables	OR (95%CI)	OR (95%CI)	OR (95%CI)
Government	1.354 (0.992–1.849)	1.492(1.053–2.115)*	1.377(0.966–1.964)
Enterprises	Reference	Reference	Reference
Male	1.377(0.963–1.969)	1.385(0.913–2.102)	0.850(0.568–1.272)
Female	Reference	Reference	Reference
Age, years			
~30	2.022(1.192–3.429)**	0.521(0.280–0.970)*	0.584(0.316–1.080)
30~	0.937(0.620–1.417)	0.451(0.264–0.770)**	0.608(0.358–1.030)
40~	0.774(0.516–1.161)	0.547(0.319–0.937)*	0.606(0.357–1.026)
50~	Reference	Reference	Reference
Education			
Junior high school	0.834(0.451–1.544)	1.213(0.558–2.637)	0.890(0.410–1.934)
High school/Technical secondary school	0.870(0.546–1.387)	1.151(0.649–2.040)	0.817(0.467–1.429)
Junior college	0.698(0.502–0.971)	1.144(0.772–1.693)	0.836(0.565–1.238)
Bachelor’s degree and above	Reference	Reference	Reference
Married	0.941(0.618–1.431)	0.709(0.444–1.132)	1.068(0.675–1.689)
Unmarried/Divorced/Widowed	Reference	Reference	Reference
Family per-capita monthly income (RMB)			
~2000	0.516(0.317–0.841)**	0.698(0.398–1.222)	0.764(0.429–1.361)
2000~	0.635(0.443–0.909)*	0.736(0.490–1.106)	0.834(0.544–1.277)
4000~	0.907(0.628–1.310)	1.232(0.804–1.889)	0.814(0.531–1.247)
6000~	Reference	Reference	Reference
Physical activity			
High	1.744(1.163–2.614)**	1.891(1.166–3.066)**	1.736(1.074–2.805)*
Moderate	1.272(0.951–1.701)	1.479(1.062–2.059)*	1.148(0.816–1.615)
Low	Reference	Reference	Reference
Smoker	0.681(0.450–1.031)	0.583(0.657–0.951)*	0.949(0.584–1.541)
Non-smoker	Reference	Reference	Reference
Passive smoker	0.686(0.517–0.910)**	0.678(0.490–0.939)*	0.616(0.443–0.856)**
Non-passive smoker	Reference	Reference	Reference
Alcohol intake	1.212(0.860–1.710)	1.084(0.728–1.616)	1.375(0.918–2.060)
Non-alcohol drinker	Reference	Reference	Reference
Health culture at individual level	1.478(1.152–1.896)**	1.654(1.256–2.179)**	2.916(2.189–3.884)**
Unhealthy behaviors of direct leadership	0.918(0.818–1.031)	0.954(0.934–1.092)	0.927(0.809–1.064)
Adverse health effects of direct leadership	0.963(0.843–1.102)	0.852(0.722–1.005)	1.002(0.852–1.178)
Beneficial health effects of direct leadership	0.974(0.789–1.202)	0.946(0.747–1.199)	1.405(1.107–1.783)**
Health culture at workplace level	1.079(0.854–1.364)	1.368(1.058–1.768)*	1.272(0.970–1.667)

*P < 0.05;

**P < 0.01

## Discussion

Although workplace health culture can be considered as an integral feature of a workplace, due to individual differences in cognition and position, we believe that workplace health culture should be measured as an individual-level variable. Therefore, we first quantitatively measured health culture using the WHCS [3]. However, the results of workplace health culture were difficult to compare with other studies because few studies quantitatively measured health culture. Thus, we mainly discussed the results of this study. Based on our findings, the ratings of three dimensions were more than 4 (out of 5) which indicating a moderate good health culture especially in dimension of health culture at individual level. Among all the five dimensions, the ratings of health culture at individual level was highest, 4.38; while the ratings of unhealthy behaviors of direct leadership was lowest, 3.74. These findings suggested that workplace health promotion should pay more attention on improving the health behavior of leadership rather than that of employees. Moreover, the leaders should recognize that their health-related behaviors will have both positive and negative impact on the employees.

Regarding the health outcomes, we found a modest level of good health-related outcomes, including good SRH (73.5%), good mental health (81.4%), and good happiness (78.2%). Compared with our results, a study in Singapore found a higher level of good or excellent SRH in 77% of respondents [23], while another study in the Chinese occupational population reported a lower proportion of good SRH [9]. Regarding mental health, Gao et al. [24] studied 2,796 employees from 35 Chinese workplaces and found a level of good mental health of 65.1%, which was obviously lower than that in our study. In terms of happiness, a study of 18,622 Americans showed that about 67% of adults had high levels of well-being. The discrepancy between our study and these reports might be due to a higher proportion of respondents from administrative offices. As our results showed, participants from administrative offices had significantly better health-related outcomes (SRH, mental health, and happiness). Despite contrasting results with the aforementioned studies, our findings were consistent with other previous studies in China [9, 25]. Therefore, it is necessary to discuss the possible reasons for these results.

Civil servants are considered to have the most stable and respectable job in China. Meanwhile, participants from enterprises might have more pressure and higher risk of unemployment than those in administrative offices. Our study indicated that age was negatively associated with good SRH, but positively associated with good mental health. These 2 findings were consistent with those of another 2 studies [11, 26]. The possible reason for this consistency might be because young employees used to have better physical health but greater pressure in lower positions, and had less income than their seniors. In addition, we also found that socioeconomic circumstances were related to SRH, which was also supported by another study [11]. However, some studies reported that health-related outcomes were associated with some other demographic variables such as gender, marital status, education, which was not found in our logistic regression results [9, 14]

Based on our findings, physical activity had the most positive impact on the 3 health-related outcomes, while passive smoking had the most negative impact. Regarding the other two health behaviors, smokers had 0.583 times lower odds of reporting good mental health. Furthermore, there were no associations between drinking alcohol and health-related outcomes. These findings suggested that physical activity and passive smoking might be important targets for workplace health promotion. In addition, improving physical activity and passive smoking might be more feasible than trying to decrease smoking and drinking.

Particularly noteworthy was the role of workplace health culture. In general, workplace health culture was positively associated with all health-related outcomes, which supported several previous qualitative studies [5, 7, 27]. Variance analyses results showed that individual health culture, beneficial health effects of direct leaders, and health culture at the workplace level were all positively associated with the 3 health-related outcomes. Unhealthy behaviors of direct leaders and adverse health effects of direct leaders were only associated with happiness. After introducing demographic variables and health behaviors, logic regression results revealed that health culture at the individual level might be most important because it was positively associated with SRH, mental health, and happiness. Regarding the level of direct leaders, scores of beneficial health effects were associated with happiness, while health culture at the workplace level was associated with mental health. Interestingly, there were no associations between health-related outcomes with the other 2 variables at the level of direct leaders. For unhealthy behaviors of direct leaders, we measured the smoking and drinking behaviors of the respondents’ direct leaders. Due to the smoke-free policy in Shanghai introduced in 2010 [28], smoking has been significantly reduced in many workplaces, particularly in administrative offices. Meanwhile, due to the Eight Regulations for civil servants [29], drinking alcohol has been also significantly reduced in administrative offices. Unless the relationship between the employee and the leader was close enough, the behavior of the leader led to a limited impact on the employee's behavior outside the workplace. Similarly, there were no associations between health-related outcomes and the adverse health effects of direct leaders, such as encouraging employees to smoke and drink, and hoping employees to work overtime.

It was worth noting that the influencing factors of different health outcomes were not identical. Therefore, if the main objective of health promotion is different, the design of interventions should also be various. For example, as a relatively comprehensive indicator, happiness was positively associated with physical activity, passive smoking, and health culture, but had nothing to do with demographic characteristics. These findings suggested that fostering a health culture and improving employee’s lifestyles should be taken seriously for employees’ happiness. More importantly, there were several of the same influencing factors on different health-related outcomes. Therefore, physical activity, passive smoking, and individual health culture might play a critical role in workplace health promotion.

There are some limitations to our study. First, all measures were based on self-reports, even though the measures have been validated [3, 18, 19, 22]. Second, the direction of causality could not be addressed due to the cross-sectional study design. Third, transforming continuous variables into categorical variables might decrease the statistical power and precision of the study. A two-level hierarchical linear model or multiple linear regression analysis should have been employed as respondents were clustered within workplaces. However, the results of testing showed that our data appeared not applicable to multi-level analysis and multiple linear regression analysis. References to the data and the results of testing are also given with the S1 Dataset and S3 File.

## Conclusions

In this paper, we found that workplace health culture, health behaviors, and demographic characteristics were associated with health-related outcomes. Furthermore, individual health culture, physical activity, and passive smoking might play a critical role in workplace health promotion.

## Supporting information

S1 File Health Workplace Survey (in Chinese). (PDF)

S2 File Health Workplace Survey (in English). (PDF)

S3 File Supplementary materials for data testing. (PDF)

S1 Dataset Data. (SAV)

## Abbreviations

FS: Flourishing Scale
IPAQ: International Physical Activity Questionnaire
OR: Odds Ratio
SRH: self-rated health
WHCS: Workplace Health Culture Scale
