# Supplementary material for: Associations between health culture, health behaviors, and health-related outcomes: A cross-sectional study
Source: PLoS One. 2017 Jul 26;12(7):e0178644. doi: 10.1371/journal.pone.0178644 (PMC5528893; doi:10.1371/journal.pone.0178644)
Supplement: S1 File — (PDF) [file pone.0178644.s001.pdf]

健康单位调查问卷

第一部分　人口学信息

（一） 基本人口特征

A1. 性别: 1)男　　　　2)女

A2. 出生日期 ________年_______月

A3. 文化程度：

1） 初中及以下　2）高中、中专及技校 3）大专　4）本科　5）硕士及以上

A4. 婚姻：1）未婚　2）已婚（在婚）　3）丧偶　4）离婚　5）其它______

A5. 家庭人均月收入：1) <800 元　2）800-2000 元　3）2000-4000 元

4）4000-6000 元　5）6000-8000 元　6）>8000 元

A6. 单位类型： 1)企业　　2）政府机关

第二部分　健康状况

B1.总的来说，您认为您的健康状况是:**（依据个人感受在以下五个选项中，选择合适的一个）**

☐非常好　☐很好　☐好　☐一般　☐差

B2.请在下面 5 个句子的描述中标出**过去 2 周**里你最接近的感觉状态。*如：您在过去 2 周有一半以上时间感到快乐、心情舒畅，就在其后 3 对应的方框内打"√"。*

过去 2 周	所有时间	大部分时间	超过一半时间	少于一半时间	有时候	从未有过
B2.1 我感觉快乐、心情舒畅	☐5	☐4	☐3	☐2	☐1	☐0
B2.2 我感觉宁静和放松	☐5	☐4	☐3	☐2	☐1	☐0
B2.3 我感觉充满活力、精力充沛	☐5	☐4	☐3	☐2	☐1	☐0
B2.4 我睡醒时感到清新、得到了充足休息	☐5	☐4	☐3	☐2	☐1	☐0
B2.5 我每天的生活充满了有趣的事情	☐5	☐4	☐3	☐2	☐1	☐0

B3. 您对以下的 8 项条目可能赞同或者不赞同。请用下面问卷中的 1-7 分的选项来评价这些条目，请在您认为符合您情况的评分的方框内打"√"。

（7：完全同意；6：同意；5：有点同意；4：既不同意也不反对；3：有点不同意；2：不同意；1：完全不同意）.

**B3. 幸福感**	**完全不同意**	**1**	**2**	**3**	**4**	**5　6　7**	**完全同意**
B3.1.我过着有追求和有意义的生活	**□1**	**□2**	**□3**	**□4**	**□5**	**□6**	**□7**
B3.2.我在我的社会关系中受人拥护和爱戴	**□1**	**□2**	**□3**	**□4**	**□5**	**□6**	**□7**
B3.3.我平日的生活中充满乐趣	**□1**	**□2**	**□3**	**□4**	**□5**	**□6**	**□7**
B3.4.我会主动帮助他人获得幸福和快乐	**□1**	**□2**	**□3**	**□4**	**□5**	**□6**	**□7**
B3.5.我能胜任对我来说重要的事务	**□1**	**□2**	**□3**	**□4**	**□5**	**□6**	**□7**
B3.6.我是个好人而且生活得很幸福	**□1**	**□2**	**□3**	**□4**	**□5**	**□6**	**□7**
B3.7.我对我的未来很乐观	**□1**	**□2**	**□3**	**□4**	**□5**	**□6**	**□7**
B3.8.人们尊重我	**□1**	**□2**	**□3**	**□4**	**□5**	**□6**	**□7**

第三部分　健康行为

C1. 到目前为止，您是否吸够 100 支烟？

☐否　　　　　☐是

C2 在过去 1 周，您有几天吸入（持续或者累计）别人吸烟产生的烟雾（15 分钟以上）？

☐有 ________天　　　　　☐没有

C3. 您通常会在哪些地方被动吸烟？(**可多选**)

☐家里　　　☐工作场所　　　☐公共交通工具及等候室

☐娱乐场所　　　☐餐厅　　　☐其他(请标明)____________

C4. 在过去 7 天中，您进行以下活动的时间：(请只记录每次至少**持续 10 分钟**的身体活动，没有请填写“0”)

身体活动类别	过去 7 天中有	平均每天
a. **重身体活动**（重身体活动是指需要您花费大力气完成，呼吸较平常明显增强的活动。)例如：	____天	_____分钟
b. **中等强度身体活动**(中等强度身体活动是指需要您花费中等力气完成，呼吸较平常稍微增强的活动)，例如：	____天	_____分钟
c. **步行**（包括您工作时和在家中的步行，交通行程的步行以及为了锻炼身体进行的步行。）	____天	_____分钟
d. **静态**（包括您在工作单位和家中，坐在办公桌前，电脑前，坐着或躺着看电视，拜访朋友，看书，乘车等的时间）	____天	_____分钟

C5. 您是否饮酒(如啤酒、葡萄酒、白酒、红酒、黄酒等)？

☐是　　　　　2）☐否

第四部分　健康文化

**D. 企业健康文化**	完全不同意 1	2	3	4	5 完全同意
**针对您本人**					
D1 采用健康的生活方式对我来说很重要	□1	□2	□3	□4	□5
D2 与健康活动可以扩大朋友圈和认识更多新人	□1	□2	□3	□4	□5
D3 工会由于采纳健康的行为方式受到表彰或重视	□1	□2	□3	□4	□5
D4 我的家庭成员或合租者支持别人采纳健康的行为生活方式，在我们公司，这些是正常的	□1	□2	□3	□4	□5
**针对您的直接领导**					

D5 我的直接领导者喜欢吸烟	□1	□2	□3	□4	□5
D6 我的直接领导者喜欢饮酒	□1	□2	□3	□4	□5
D7 我的直接领导者喜欢锻炼身体	□1	□2	□3	□4	□5
D8 我的直接领导者会劝我吸烟	□1	□2	□3	□4	□5
D9 我的直接领导者会劝我饮酒	□1	□2	□3	□4	□5
D10 我的直接领导者希望我加班	□1	□2	□3	□4	□5
D11 我的直接领导者会鼓励我锻炼身体	□1	□2	□3	□4	□5
D12 我的直接领导支持员工采纳健康的行为生活方式	□1	□2	□3	□4	□5
D13 我的直接领导相互支持采纳健康的行为生活方式	□1	□2	□3	□4	□5
**针对公司整体**					
D14 公司通过提供时间、地点和资金来支持健康项目	□1	□2	□3	□4	□5
D15 我们公司教育员工要有健康的生活方式	□1	□2	□3	□4	□5
D16 公司新员工注意到公司对健康行为方式的支持	□1	□2	□3	□4	□5
D17 不健康的行为方式（如吸烟和酗酒）不被鼓励	□1	□2	□3	□4	□5
D18 在我们公司，人们具有团队精神（如，人们真的了解别人，有归属感，当别人有需要时能及时提供帮助）	□1	□2	□3	□4	□5
D19 我们公司有共识（例如，人们感到企业的工作是与他们的个人价值观是一致的）	□1	□2	□3	□4	□5
D20 我们公司有积极向上的理念（例如：在享受工作，庆祝成功，接受“我能做到”的观点）	□1	□2	□3	□4	□5
