# Supplementary material for: Associations between health culture, health behaviors, and health-related outcomes: A cross-sectional study
Source: PLoS One. 2017 Jul 26;12(7):e0178644. doi: 10.1371/journal.pone.0178644 (PMC5528893; doi:10.1371/journal.pone.0178644)
Supplement: S2 File — (PDF) [file pone.0178644.s002.pdf]

Health Workplace Survey

Part 1 Demographic Characteristics

A1. Gender:　1) Male　　　　　　2) Female

A2. Date of birth:　______________

A3. Education：

1) Junior high school　2）High School/ technical secondary school　3）Junior college

4）Bachelor　5）Master's or higher degree

A4. Marital status：1）Unmarried　2）Married　3）Widowed　4）Divorced

5）Other______

A5. Family per-capita monthly income (RMB)：

1)<800　2）800-2000　3）2000-4000　4）4000-6000　5）6000-8000　6）>8000

A6. Type of workplace：1) Enterprises　　2）Government

Part 2 Health-related Outcomes

B1. Would you say that in general your health is?

☐Perfect　☐very good　☐Good　☐Fair　☐Poor

B2.Please indicate for each of the five statements which is closest to how you have been feeling over the last two weeks. Notice that higher numbers mean better well-being.

Example: If you have felt cheerful and in good spirits more than half of the time during the last two weeks, put a tick in the box with the number 3 in the upper right corner.

Over the last two weeks	All of the time	Most of the time	More than half of the time	Less than half of the time	Some of the time	At no time
B2.1 I have felt cheerful and in good spirits	□5	□4	□3	□2	□1	□0
B2.2 I have felt calm and relaxed	□5	□4	□3	□2	□1	□0
B2.3 I have felt active and vigorous	□5	□4	□3	□2	□1	□0
B2.4 I woke up feeling fresh and rested	□5	□4	□3	□2	□1	□0
B2.5 My daily life has been filled with things that interest me	□5	□4	□3	□2	□1	□0

B3. Below are eight statements with which you may agree or disagree. Using the 1-7 scale below., indicate your agreement with each item by indicating that response for each statement.

7. Strongly agree　6. Agree　5. Slightly agree　4. Mixed or neither agree nor disagree

3. Slightly disagree　2. Disagree　1. Strongly disagree

**B3.Flourishing Scale**	Strongly disagree 1	2	3	4	5	6	7 Strongly agree
B3.1 I lead a purposeful and meaningful life	□1	□2	□3	□4	□5	□6	□7
B3.2 My social relationships are supportive and rewarding	□1	□2	□3	□4	□5	□6	□7
B3.3 I am engaged and interested in my daily activities	□1	□2	□3	□4	□5	□6	□7
B3.4 I actively contribute to the happiness and well-being of others	□1	□2	□3	□4	□5	□6	□7
B3.5 I am competent and capable in the activities that are important to me	□1	□2	□3	□4	□5	□6	□7
B3.6 I am a good person and live a good life	□1	□2	□3	□4	□5	□6	□7
B3.7 I am optimistic about my future	□1	□2	□3	□4	□5	□6	□7
B3.8 People respect me	□1	□2	□3	□4	□5	□6	□7

Part 3 Health-related Behaviors

C1. Have you smoked for more than 100 cigarettes？

□no □yes

C2 In the past week, have you exposed to (sustained or cumulative) smoke for 15 minutes or more？

□yes________days □none

C3. In the past week，please indicate your physical activity:（Only report physical activity for 10 minutes or more）

Physical activity	In the last 7 days	Average time every day
**a.Heavy physical activity** (refers to activities that require you to spend a lot of effort and breathe more than usual). For example:	____Days	_____Min
**b.Moderate physical activity** (refers to activities that require you to spend moderate effort and breathe slightly more usual). For example:	____Days	_____Min
**c.Walking**（including your work and walking at home, walking on footsteps and walking for exercise).	____Days	_____Min
**d.Sedentary behavior** (including working at home, sitting at the desk, before the computer, sitting or lying for watching TV, visiting friends, reading, traveling, etc.)	____Days	_____Min

C4. Do you drink alcohol (such as beer, wine, white wine, red wine, rice wine, etc.)?

□yes □no

Part 4 Health culture

**Workplace health culture**	Strongly disagree 1	2	3	4	5 Strongly agree
Individual					
D1 It is important for me to lead a healthy lifestyle	□1	□2	□3	□4	□5
D2 Participation in health activities can enlarge my circle of friends	□1	□2	□3	□4	□5
D3 Employee will be commended and paid attention to due to healthy behaviors	□1	□2	□3	□4	□5
D4 My family or roommates support me to lead a healthy lifestyle	□1	□2	□3	□4	□5
Direct leadership					
D5 My direct leaders like smoking	□1	□2	□3	□4	□5
D6 My direct leaders like drinking	□1	□2	□3	□4	□5
D7 My direct leaders encourage me to smoke	□1	□2	□3	□4	□5
D8 My direct leaders encourage me to drink	□1	□2	□3	□4	□5
D9 My direct leaders hope that I can work overtime	□1	□2	□3	□4	□5
D10 My direct leaders like to exercise	□1	□2	□3	□4	□5
D11 My direct leaders encourage me to exercise	□1	□2	□3	□4	□5
D12 My direct leaders encourage me to lead a healthy lifestyle	□1	□2	□3	□4	□5
D13 My direct leaders support each other to lead a healthy lifestyle	□1	□2	□3	□4	□5
Workplace					
D14 Resources are provided to support health promotion	□1	□2	□3	□4	□5
D15 Employee are taught to lead a healthy lifestyle	□1	□2	□3	□4	□5
D16 New employee notice organizational support on health behaviors	□1	□2	□3	□4	□5
D17 Unhealthy behaviors are not encouraged	□1	□2	□3	□4	□5

D18 People have team spirit	□1	□2	□3	□4	□5
D19 A consensus has been reached	□1	□2	□3	□4	□5
D20 People have positive perception	□1	□2	□3	□4	□5
