# Supplementary material for: Associations between health culture, health behaviors, and health-related outcomes: A cross-sectional study
Source: PLoS One. 2017 Jul 26;12(7):e0178644. doi: 10.1371/journal.pone.0178644 (PMC5528893; doi:10.1371/journal.pone.0178644)
Supplement: S3 File — (PDF) [file pone.0178644.s003.pdf]

Supplementary materials for data testing

1. Test for multilevel model

There should be a two-level hierarchical linear model with people clustered within workplaces. We had tested whether the data is applicable to multi-level analysis by SAS.9.3. The results were as follows.

(1) For self-rated health, we found that ICC(intraclass correlation coefficient)=0.1907/(0.1907+3.29) =0.054788. Among all the 16 workplaces, there were only two significant random effect.

Parameter Estimation of Covariance (SRH as a dependent variable)			
Covariance parameter	Variable	Parameter Estimation	Standard error
Intercept	Workplace	0.004624	0.002459
Residual		0.1907	0.007003

Fixed effect(SRH as a dependent variable)					
Effect	Parameter Estimation	Standard error	Degrees of freedom	t	P
Intercept	0.7335	0.02051	15	35.75	<0.0001

Random effect(SRH as a dependent variable)					
Effect	Variable	Parameter Estimation	Std Err Pred	t	P
Intercept	Workplace 1	-0.01073	0.0392	-0.27	0.7844
Intercept	Workplace 2	0.04614	0.03824	1.21	0.2278
Intercept	Workplace 3	0.0218	0.03815	0.57	0.5678
Intercept	Workplace 4	-0.0336	0.03931	-0.85	0.3927
Intercept	Workplace 5	0.0274	0.03714	0.74	0.4608
Intercept	Workplace 6	0.08892	0.03746	2.37	**0.0177**
Intercept	Workplace 7	0.0469	0.04369	1.07	0.2832
Intercept	Workplace 8	-0.04996	0.04454	-1.12	0.2621
Intercept	Workplace 9	0.004276	0.03995	0.11	0.9148
Intercept	Workplace 10	0.08256	0.04471	1.85	0.065
Intercept	Workplace 11	0.004623	0.03951	0.12	0.9069
Intercept	Workplace 12	-0.1327	0.03962	-3.35	**0.0008**
Intercept	Workplace 13	-0.05401	0.03995	-1.35	0.1765
Intercept	Workplace 14	0.01899	0.04088	0.46	0.6423
Intercept	Workplace 15	-0.00996	0.04507	-0.22	0.8252
Intercept	Workplace 16	-0.05064	0.04088	-1.24	0.2156

(2) For mental health, we found that ICC(intraclass correlation coefficient)=0.1469/(0.1469+3.29) =0.042742. Among all the 16 workplaces, there were only two significant random effect.

Parameter Estimation of Covariance (Mental health as a dependent variable)			
Covariance parameter	Variable	Parameter Estimation	Standard error
Intercept	Workplace	0.005294	0.002565
Residual		0.1469	0.005406

Fixed effect(Mental health as a dependent variable)					
Effect	Parameter Estimation	Standard error	Degrees of freedom	t	P
Intercept	0.8115	0.02081	15	38.99	<0.0001

Random effect(Mental health as a dependent variable)					
Effect	Variable	Parameter Estimation	Std Err Pred	t	P
Intercept	Workplace 1	0.07205	0.03731	1.93	0.0537
Intercept	Workplace 2	-0.04544	0.03664	-1.24	0.2151
Intercept	Workplace 3	0.06612	0.03637	1.82	0.0693
Intercept	Workplace 4	-0.00613	0.03692	-0.17	0.8682
Intercept	Workplace 5	0.05045	0.0353	1.43	0.1532
Intercept	Workplace 6	0.04581	0.0357	1.28	0.1996
Intercept	Workplace 7	-0.0332	0.04186	-0.79	0.4279
Intercept	Workplace 8	0.0207	0.04275	0.48	0.6283
Intercept	Workplace 9	0.01697	0.03805	0.45	0.6556
Intercept	Workplace 10	0.1089	0.04275	2.55	**0.011**
Intercept	Workplace 11	0.02506	0.03805	0.66	0.5103
Intercept	Workplace 12	-0.04392	0.03783	-1.16	0.2459
Intercept	Workplace 13	-0.1392	0.03816	-3.65	**0.0003**
Intercept	Workplace 14	-0.00863	0.03936	-0.22	0.8264
Intercept	Workplace 15	-0.07621	0.04332	-1.76	0.0787
Intercept	Workplace 16	-0.05327	0.03886	-1.37	0.1706

(3) For happiness, we found that ICC(intraclass correlation coefficient)=0.1607/(0.1607+3.29) =0.04657. Among all the 16 workplaces, there were only three significant random effect

Parameter Estimation of Covariance (Happiness as a dependent variable)			
Covariance parameter	Variable	Parameter Estimation	Standard error
Intercept	Workplace	0.01087	0.004647
Residual		0.1607	0.005925

Fixed effect(Happiness as a dependent variable)					
Effect	Parameter Estimation	Standard error	Degrees of freedom	t	P
Intercept	0.7833	0.02815	15	27.83	<0.0001

Random effect(Happiness as a dependent variable)					
Effect	Variable	Parameter Estimation	Std Err Pred	t	P
Intercept	Workplace 1	0.07066	0.04439	1.59	0.1116
Intercept	Workplace 2	-0.00573	0.04342	-0.13	0.8951
Intercept	Workplace 3	0.08209	0.04332	1.89	0.0583
Intercept	Workplace 4	-0.01873	0.04416	-0.42	0.6716
Intercept	Workplace 5	-0.01202	0.04214	-0.29	0.7756
Intercept	Workplace 6	0.09651	0.04266	2.26	**0.0238**
Intercept	Workplace 7	0.06931	0.04976	1.39	0.1638
Intercept	Workplace 8	0.08554	0.05085	1.68	0.0927
Intercept	Workplace 9	-0.03786	0.04523	-0.84	0.4027
Intercept	Workplace 10	0.1241	0.05085	2.44	**0.0148**
Intercept	Workplace 11	-0.01769	0.0451	-0.39	0.695
Intercept	Workplace 12	-0.2815	0.04548	-6.19	**<0.0001**
Intercept	Workplace 13	-0.06612	0.04575	-1.45	0.1486
Intercept	Workplace 14	-0.00142	0.04645	-0.03	0.9756
Intercept	Workplace 15	-0.07065	0.05181	-1.36	0.1729
Intercept	Workplace 16	-0.01649	0.04616	-0.36	0.7211

(4) In summary, the results of testing showed that our data appeared not applicable to multi-level analysis. First, all the ICC were small. Second, the difference between the workplace level was very small and almost not statistical significant.

2. Test for multiple linear regression

We had tested whether the data is applicable to multiple linear regression analysis by SAS.9.3. The results were as follows.

(1) For SRH: figure1 showed the variance nonhomogeneity of residual; figure 2 showed that SRH was close to normal distribution by normal distribution test; the other five figures showed that there was no significant linear correlation between the five variables of health culture and self-rated health.

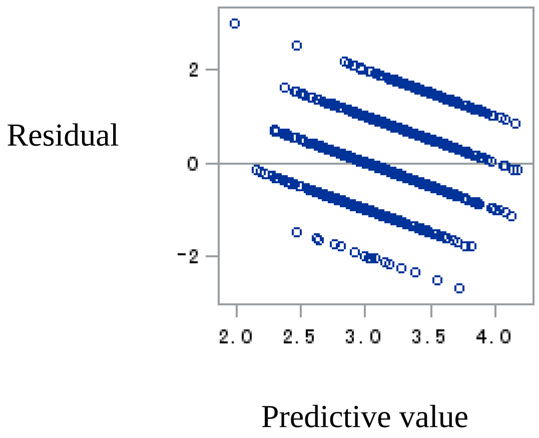

Figure 1. Test for Variance homogeneity of Residual

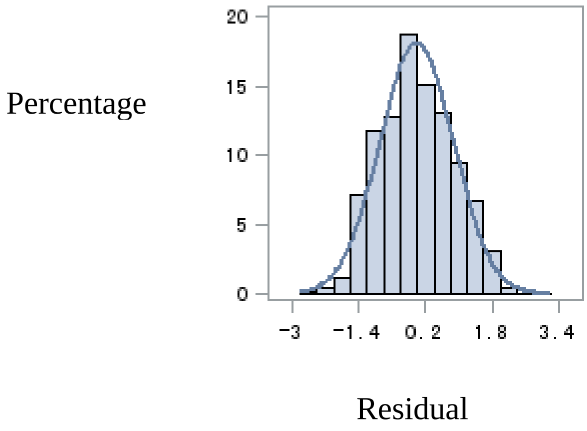

Figure 2. Test for Normality

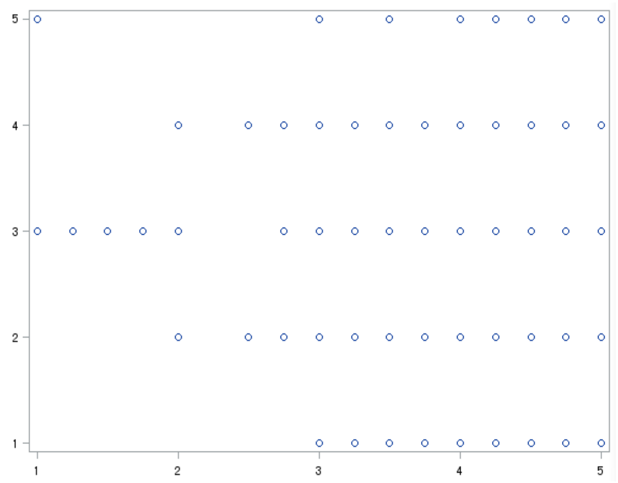

Figure 3. Scatter plot for SRH and Individual health culture

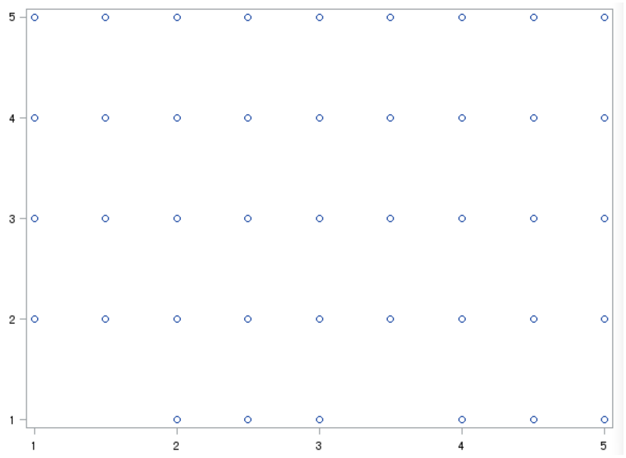

Figure 4. Scatter plot for SRH and Adverse health behaviors of direct leaders

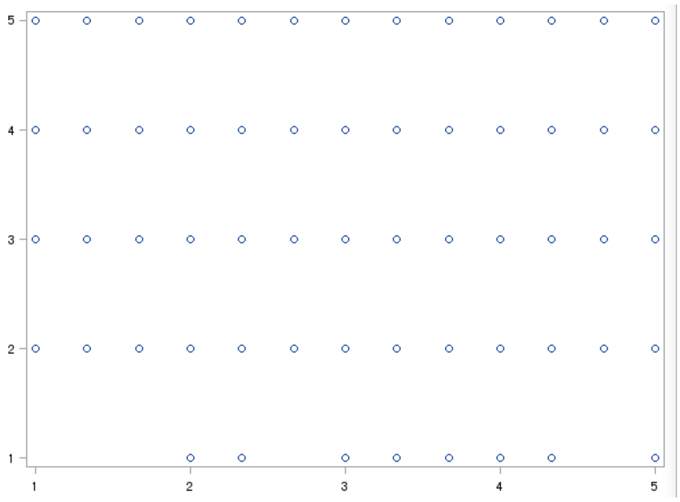

Figure 5. Scatter plot for SRH and Adverse health effects of direct leaders

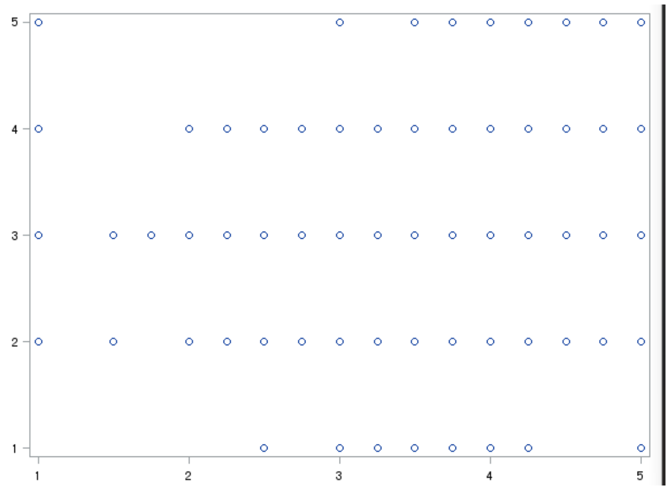

Figure 6. Scatter plot for SRH and Beneficial health effects of direct leaders

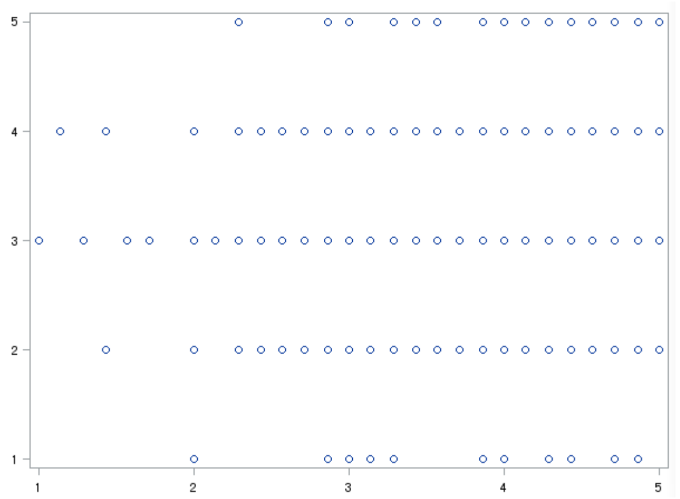

Figure 7. Scatter plot for SRH and Overall health culture

(2) For mental health: figure 8 showed the variance nonhomogeneity of residual; figure 9 showed that mental health was not close to normal distribution by normal distribution test; the other five figures showed that there was no significant linear correlation between the five variables of health culture and score of mental health.

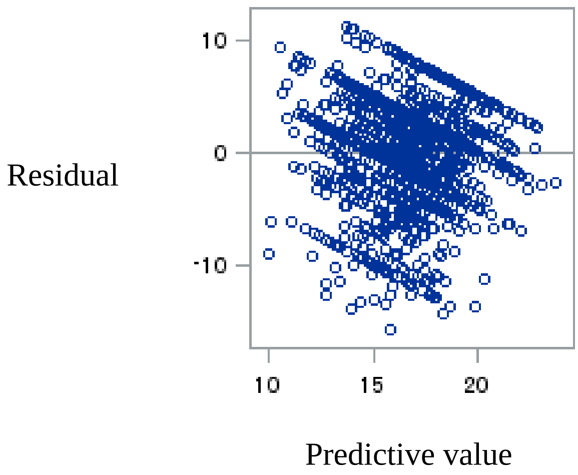

Figure 8. Test for Variance homogeneity of Residual

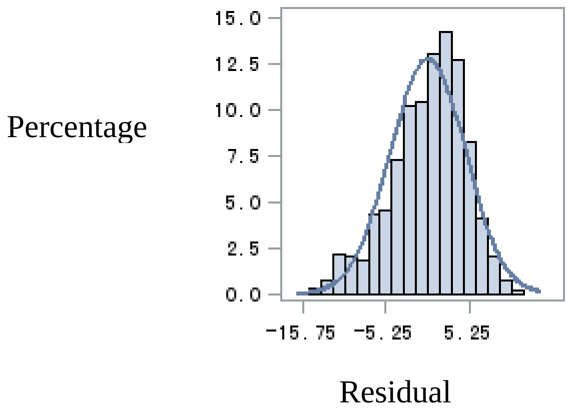

Figure 9. Test for Normality

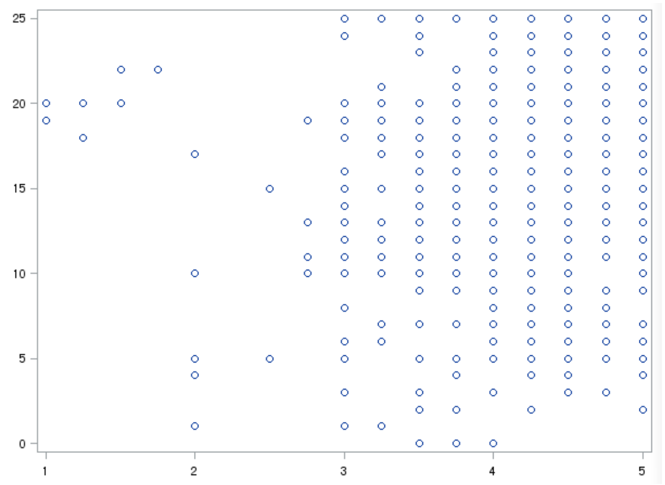

Figure 10. Scatter plot for mental health and individual health culture

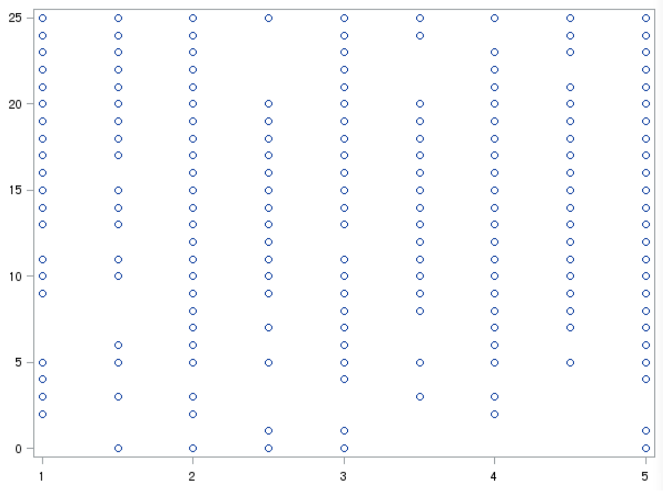

Figure 11. Scatter plot for mental health and adverse health behaviors of direct leaders

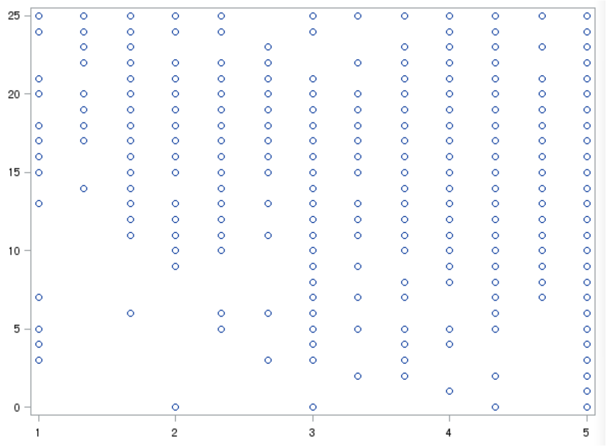

Figure 12. Scatter plot for mental health and adverse health effects of direct leaders

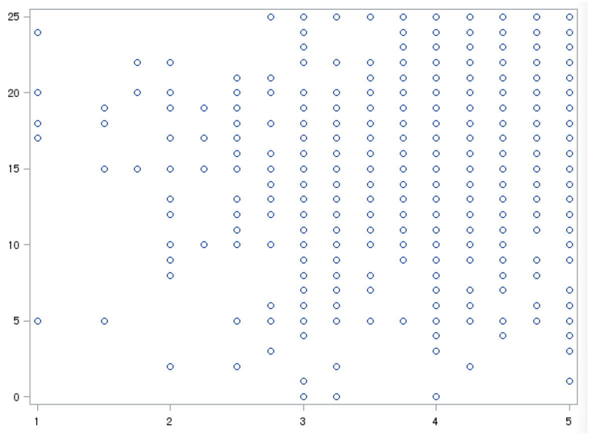

Figure 13. Scatter plot for mental health and beneficial health effects of direct leaders

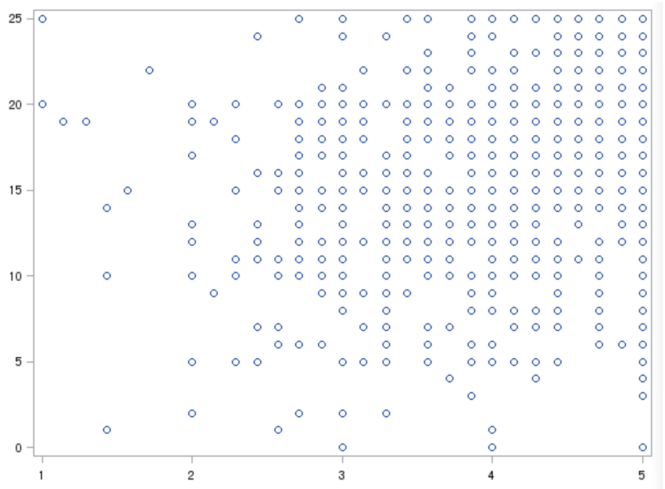

Figure 14. Scatter plot for mental health and Overall health culture

(3) For happiness: figure 15 showed the variance nonhomogeneity of residual; figure 16 showed that happiness was not close to normal distribution by normal distribution test; the other five figures showed that there was no significant linear correlation between the five variables of health culture and score of happiness.

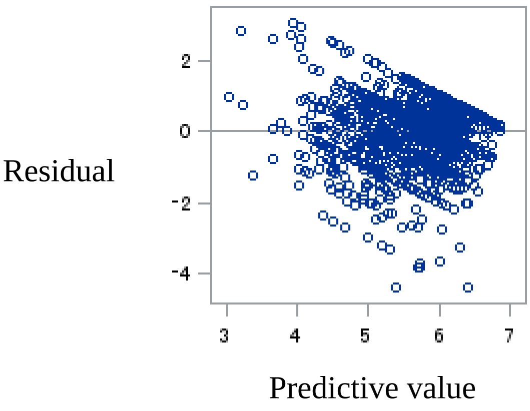

Figure 15. Test for Variance homogeneity of Residual

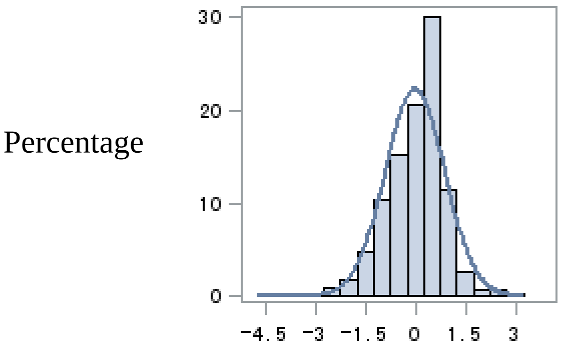

Figure 16. Test for Normality

Residual

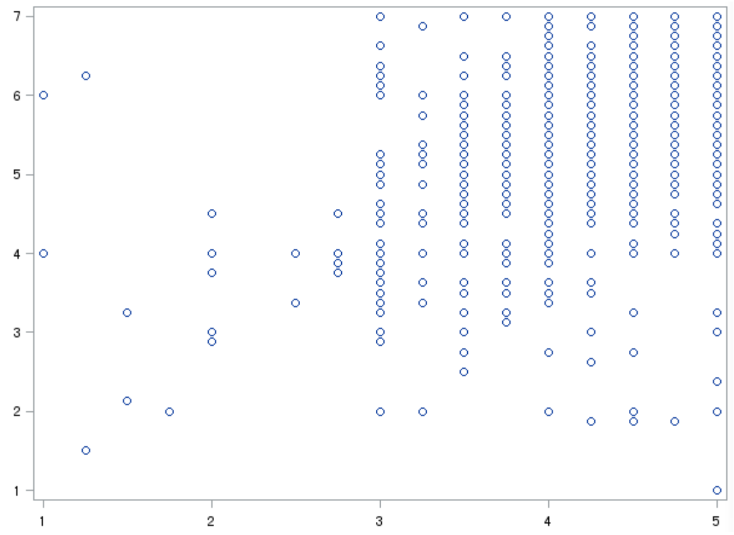

Figure 17. Scatter plot for happiness and individual health culture

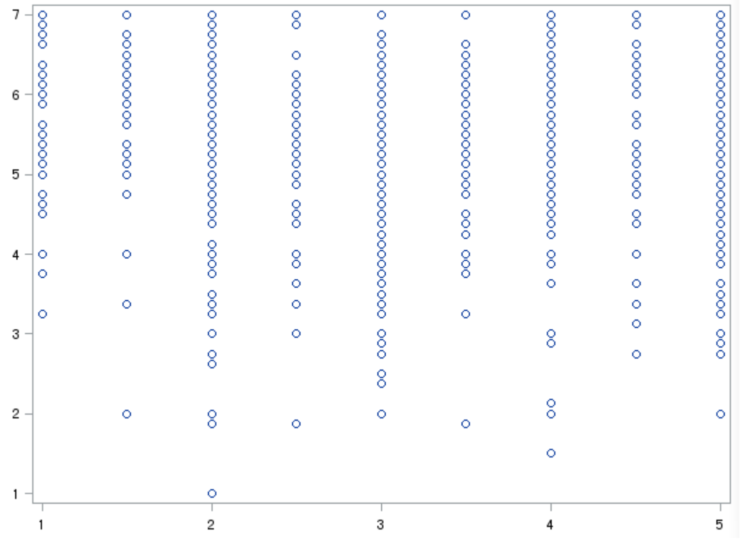

Figure 18. Scatter plot for happiness and adverse health behaviors of direct leaders

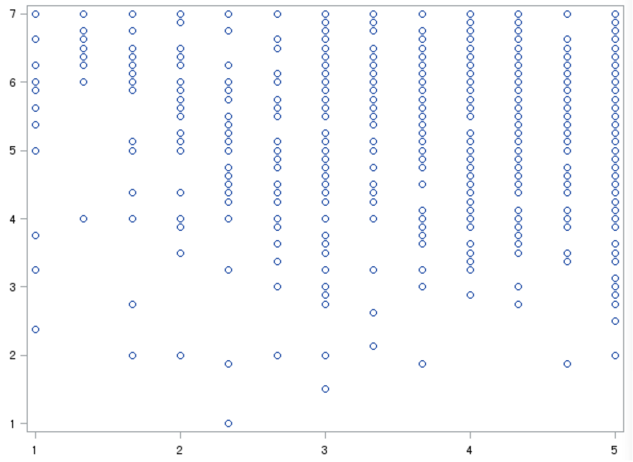

Figure 19. Scatter plot for happiness and adverse health effects of direct leaders

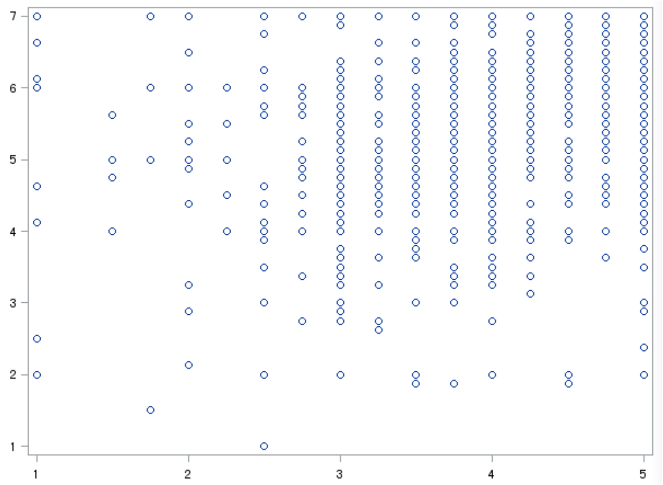

Figure 20. Scatter plot for happiness and beneficial health effects of direct leaders

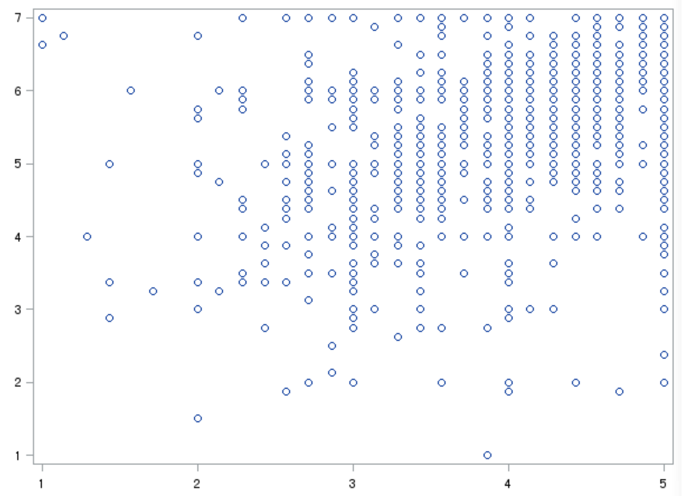

Figure 21. Scatter plot for happiness and Overall health culture

(4) In summary, the results of testing showed that our data appeared not applicable to multiple linear regression analysis due to the variance nonhomogeneity of residual and no significant linear correlation.
